# 合并重症肌无力的胸腺瘤患者术后生存的初步分析——ChART数据库回顾性结果

**DOI:** 10.3779/j.issn.1009-3419.2016.07.03

**Published:** 2016-07-20

**Authors:** 昉睿 汪, 烈文 庞, 剑华 傅, 毅 沈, 煜程 魏, 黎杰 谭, 鹏 张, 泳涛 韩, 椿 陈, 仁泉 张, 印 李, 克能 陈, 和忠 陈, 永煜 刘, 有斌 崔, 允 王, 振涛 于, 鑫明 周, 阳春 柳, 媛 刘, 志涛 谷, 文涛 方, 中国胸腺肿瘤协作组成员

**Affiliations:** 1 200032 上海，复旦大学附属华山医院胸外科 Department of Thoracic Surgery, Huashan Hospital, Fudan University, Shanghai 200040, China; 2 510060 广州，中山大学附属肿瘤医院胸外科 Department of Thoracic Surgery, Guangdong Esophageal Cancer Institute, Sun Yat-sen University Cancer Center, State Key Laboratory of Oncology in South China, Collaborative Innovation Center of Cancer Medicine, Guangzhou 510060, China; 3 266001 青岛，青岛大学医学院附属医院胸外科 Department of Thoracic Surgery, Affiliated Hospital of Qingdao University, Qingdao 266001, China; 4 200032 上海，复旦大学附属中山医院胸外科 Department of Thoracic Surgery, Zhongshan Hospital, Fudan University, Shanghai 200032, China; 5 300052 天津，天津医科大学附属总医院胸外科 Department of Endocrinology, Tianjin Medical University General Hospital, Tianjin 300052, China; 6 610041 成都，四川省肿瘤医院胸外科 Department of Thoracic Surgery, Sichuan Cancer Hospital, Chengdu 610041, China; 7 350001 福州，福建医科大学附属协和医院胸外科 Department of Thoracic Surgery, Fujian Medical University Union Hospital, Fuzhou 350001, China; 8 230022 合肥，安徽医科大学附属第一医院胸外科 Department of Thoracic Surgery, First Affiliated Hospital of Anhui Medical University, Hefei 230022, China; 9 450008 郑州，郑州大学附属肿瘤医院胸外科 Department of Thoracic Surgery, Affiliated Cancer Hospital of Zhengzhou University, Zhengzhou 450008, China; 10 100142 北京，北京大学附肿瘤医院胸外科 Department of Thoracic Surgery, Beijing Cancer Hospital, Beijing 100142, China; 11 200433 上海，长海医院胸心外科 Department of Cardiothoracic Surgery, Changhai Hospital, Shanghai 200433, China; 12 110042 沈阳，辽宁肿瘤医院胸外科 Department of Thoracic Surgery, Liaoning Cancer Hospital, Shenyang 110042, China; 13 130021 长春，吉林大学附属第一医院胸外科 Department of Thoracic Surgery, First Affiliated Hospital of Jilin University, Changchun 130021, China; 14 610041 成都，四川大学华西医院胸外科 Department of Thoracic Surgery, West China Hospital, Sichuan University, Chengdu 610041, China; 15 300060 天津，天津医科大学附属肿瘤医院食管癌中心 Department of Esophageal Cancer, Tianjin Cancer Hospital, Tianjin 300060, China; 16 310022 杭州，浙江省肿瘤医院胸外科 Department of Thoracic Surgery, Zhejiang Cancer Hospital, Hangzhou 310022, China; 17 330006 南昌，江西省人民医院胸外科 Department of Thoracic Surgery, Jiangxi People's Hospital, Nanchang 330006, China; 18 200030 上海，上海交通大学附属上海胸科医院 Department of Thoracic Surgery, Shanghai Chest Hospital, Shanghai Jiao Tong University, Shanghai 200030, China

**Keywords:** 胸腺瘤, 重症肌无力, 生存率, Thymoma, Myasthenia gravis (MG), Survival

## Abstract

**背景与目的:**

重症肌无力（myasthenia gravis, MG）对胸腺瘤患者预后的影响至今尚不明确，本文旨在比较单纯胸腺瘤与合并肌无力胸腺瘤患者的手术预后。

**方法:**

1992年至2012年中国胸腺协作组（Chinese Alliance for Research in Thymomas, ChART）数据库录入的18个胸外科中心诊断胸腺瘤并接受相关手术的患者分为合并重症肌无力组（合并组）和单纯胸腺瘤组（对照组）。收集两组患者的人口学资料及临床资料，比较两组患者生存率。

**结果:**

共1, 850例患者纳入研究，其中合并肌无力组及单纯胸腺瘤组分别421人和1429人，行胸腺全切的比例分别是91.2%和71.0%（*P* < 0.05）；肌无力组患者的WHO病理类型多分布于AB、B1和B2型，优于单纯胸腺瘤组（*P* < 0.05）；合并肌无力组的Masaoka分期较早（Ⅰ和Ⅱ期）的比例高于单纯胸腺瘤组。5年和10年的总体生存率在MG组和非MG组中分别为93%和88%; 83%和81%（*P*=0.034）；在Masaoka Ⅲ、Ⅳa和Ⅳb期胸腺瘤患者中，合并肌无力患者的生存曲线高于单纯胸腺瘤患者（*P*=0.003）。在进展型胸腺瘤患者中，MG组和非MG组患者的Masaoka Ⅲ、Ⅳa、Ⅳb的构成比相似，组织学结果中，MG组的AB/B1/B2/B3型的比例高于C型比例更高的非MG组（*P* < 0.001）。整体的单因素分析结果提示，MG、WHO分型、Masaoka分期、手术方式、化疗、放疗和临床切除状况均为预后的影响因素。而在多因素分析中，WHO分型、Masaoka分期和临床切除状况是独立的预后预测指标。

**结论:**

虽然重症肌无力不是独立的预后影响因素，但是在胸腺瘤患者中，合并MG的患者预后较优，尤其是Masaoka分期晚期的患者，可能与疾病的早期发现、病理类型分布相对较好、整体R0切除率较高以及复发率较低有关。

## Abstract (en)

**Background and objective:**

It is so far not clear that how myasthenia gravis (MG) affected the prognosis of thymoma patients. The aim of this assay is to compare the postoperative survival between patients with thymoma only and those with both thymoma and MG.

**Methods:**

The Chinese Alliance for Research in Thymomas (ChART) registry recruited patients with thymoma from 18 centers over the country on an intention to treat basis from 1992 to 2012. Two groups were formed according to whether the patient complicated MG. Demographic and clinical data were reviewed, Patients were followed and their survival status were analyzed.

**Results:**

There were 1, 850 patients included in this study, including 421 with and 1, 429 without MG. Complete thymectomy were done in 91.2% patients in MG group and 71.0% in non-MG group (*P* < 0.05). There were more percentage of patients with the histology of thymoma AB, B1, or B2 (*P* < 0.05) in MG group, and more percentage of patients with MG were in Masaoka stage Ⅰ and Ⅱ. The 5 year and 10 year OS rates were both higher in MG group (93% *vs* 88%; 83% *vs* 81%, *P*=0.034) respectively. The survival rate was significantly higher in patients with MG when the Masaoka staging was Ⅲ/Ⅳ (*P*=0.003). Among patients with advanced stage thymoma (stage Ⅲ, Ⅳa, Ⅳb), the constituent ratios of Ⅲ, Ⅳa, Ⅳb were similar between MG and Non-MG group. Histologically, however, there were significantly more proportion of AB/B1/B2/B3 in the MG group while there were more C in the non-MG group (*P*=0.000). Univariate analyses for all patients showed that MG, WHO classification, Masaoka stage, surgical approach, chemotherapy and radiotherapy and resectability were significant factors, and multivariate analysis showed WHO Classification, Masaoka stage, and resectability were strong independent prognostic indicators.

**Conclusion:**

Although MG is not an independent prognostic factor, the survival of patients with thymoma was superior when MG was present, especially in late Masaoka stage patients. Possible reasons included early diagnosis of the tumor, better histologic types, an overall higher R0 resection and less recurrence.

由于胸腺瘤的免疫学特性，有相当一部分的胸腺瘤患者并发自身免疫性疾病，其中重症肌无力（myasthenia gravis, MG）是最重要也是最常见的，其发生率在10%-45%。既往可能由于围手术期并发症的处理经验欠缺，合并重症肌无力成为胸腺瘤患者预后的不良因素^[1-4]^，而近些年来，文献^[1-3]^报道提示MG不再对胸腺瘤的预后产生负面影响，甚至成为预后的良性因素。然而重症肌无力对胸腺瘤患者术后预后的影响至今尚不明确。ChART数据库搜集了近20年全国18个临床中心的回顾性资料，本文比较了单纯胸腺瘤与合并肌无力胸腺瘤患者的术后生存以初步探讨重症肌无力对胸腺瘤疾病预后的影响。

## 1 病例资料与方法

### 1.1 病例资料

ChART数据库收集了自1992年-2012年间全国18个临床中心的回顾性资料，包括相关患者的人口学资料和临床资料。本研究中，将研究对象分为两组：临床合并重症肌无力组（合并组）和单纯胸腺瘤组（对照组）。对两组患者所有的人口学资料和临床资料均进行了比较。

本文以Masaoka-Koga分期作为最终的病理分期标准，应用WHO分型进行组织学分型。临床切除状态根据手术切除范围及病理结果分为：R0（无肿瘤残余）、R1（显微镜下肿瘤残余）、R2（大体肿瘤残余）以及活检术；并对胸腺切除程度进行分类：胸腺全切术和胸腺部分切除（切除肿瘤及部分胸腺组织）；同时收集了放化疗相关的数据。

### 1.2 统计学方法

采用SPSS软件14.0版进行统计学处理，计量资料以均数±标准差表示，采用*t*检验；计数资料行χ^2^检验。采用*Kaplan-Meier*法及*Cox*回归模型分析预后的影响因素，生存曲线的比较采用*Log-rank*检验。*P* < 0.05为差异有统计学意义。

## 2 结果

患者的基本资料见表 1，ChART数据库录入了2, 306例患者的数据，剔除了以下病例：49例仅活检者，152例缺少外科手术资料，124例缺少WHO分型，118例缺少Masaoka分级，最终共1, 850例纳入本研究，包括421例合并重症肌无力（MG组），1, 429例单纯胸腺瘤（非MG组）。MG组中女性比例高于非MG组（*P*= 0.034）；MG组患者的平均年龄较小（49 yr *vs* 52 yr, *P* < 0.001）；MG组整体生存率优于非MG组（95.95% *vs* 92.29%, *P*=0.026）；MG组的肿瘤直径较小（5.6 cm *vs* 7.2 cm, *P* < 0.001）；MG组中组织学类型分布于A和B型者多于非MG组（*P* < 0.001），且MG组中Masaoka Ⅰ和Ⅱ期者较多（*P*=0.004）。MG组中更高比例的患者接受了R0切除（90.0% *vs* 85.1%, *P*=0.009）和胸腺全切术（91.2% *vs* 71.0%, *P* < 0.0 01）。非MG组接受术后辅助化疗（8.8% *vs* 23.4%, *P* < 0.001）和术后辅助放疗（41.0% *vs* 47.1%, *P*=0.03）的患者比例高于MG组。

**1:** 两组胸腺瘤患者基本情况比较 Patients' characteristics

	MG(*n* =421)	Non-MG(*n*=1, 429)	*P*
Gender			0.034
Male	206 (48.9%)	782 (54.7%)	
Female	215 (51.1%)	647 (45.3%)	
Age (mean, yr)	49	52	< 0.001
Overall survival	95.95%	92.29%	0.026
WHO Classification			< 0.001
A	18 (4.3%)	89 (6.2%)	
AB	80 (19.0%)	356(24.9%)	
B1	68 (16.2%)	164(11.5%)	
B2	128 (30.4%)	169 (11.8%)	
B3	107 (25.4%)	256 (17.9%)	
C	19 (4.5%)	351(24.6%)	
Carcinoid	1(0.2%)	44 (3.1%)	
WHO classification			< 0.001
Type A thymoma	18 (4.3%)	89 (6.2%)	
Type B (including AB) thymoma	383 (91.0%)	945 (66.1%)	
Thymic cancer	20 (4.8%)	395 (27.6%)	
Thymectomy			< 0.001
Incomplete	37 (8.8%)	412(29.0%)	
Complete	382 (91.2%)	1, 008 (71.0%)	
Tumor size (cm)	5.6	7.2	< 0.001
Masaoka staging			0.004
Ⅰ	196 (46.6%)	547 (38.3%)	
Ⅱ	83 (19.7%)	277 (19.4%)	
Ⅲ	116 (27.6%)	458 (32.1%)	
Ⅳ	26 (6.2%)	147(10.3%)	
Chemotherapy (given)	36(8.8%)	317 (23.4%)	＜0.001
Radiotherapy (given)	167 (41.0%)	636 (47.1%)	0.031
Resectability (rate of R0 resection)	379 (90.0%)	1, 212 (85.1%)	0.009
MG: myasthenia gravis; WHO: World Health Organization. 注：本表得到版权所有者©2011-2016 Journal of Thoracic Disease复制许可。			

MG组的5年和10年总体生存率都高于非MG组（93% *vs* 88%; 83% *vs* 81%, *P*=0.034）（图 1-图 4）。当Masaoka分期是Ⅰ期时，非MG组生存率优于MG组（*P* < 0.0 01），然而当Masaoka分期是Ⅲ/Ⅳ期时，MG组的生存率则优于非MG组（*P*=0.003）。

**1:**
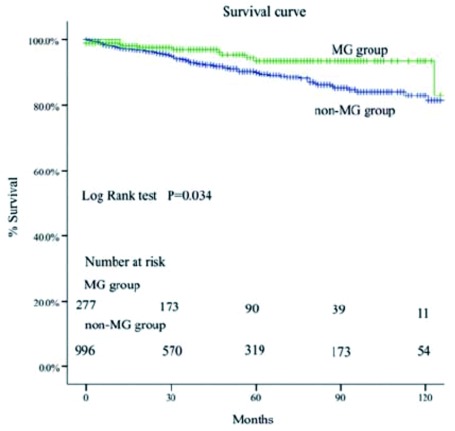
两组患者的总体生存曲线 Comparison of overall survival between myasthenia gravis (MG) group and non-MG group

**2:**
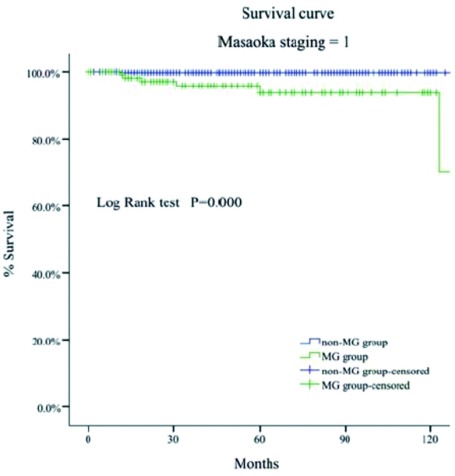
Masaoka Ⅰ期时两组患者的生存曲线 Comparison of overall survival between myasthenia gravis (MG)group and non-MG group in Masaoka stage Ⅰ

**3:**
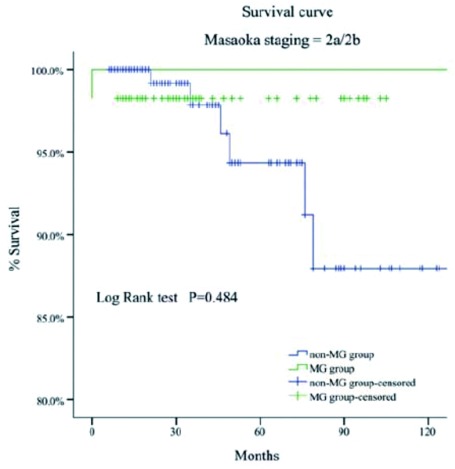
Masaoka Ⅱa/Ⅱb期两组患者的生存曲线 Comparison of overall survival between myasthenia gravis (MG)group and non-MG group in Masaoka stage Ⅱ

**4:**
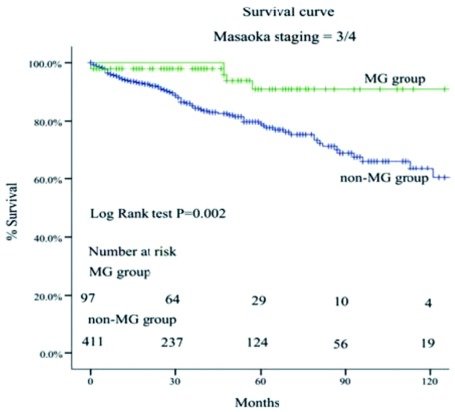
Masaoka Ⅲ/Ⅳ期两组患者的生存曲线 Comparison of overall survival between myasthenia gravis (MG) group and non-MG group in Masaoka stage Ⅲ and Ⅳ

当Masaoka分期为Ⅲ期、Ⅳa期和Ⅳb期时（表 2），两组患者的Ⅲ期和Ⅳ期构成比相似。但在组织学类型上，MG组的A B/B1/B2/B3型的比例较高，而非MG组C型比例较高（*P* < 0.001）。MG组的肿瘤直径较小（6.4 cm *vs* 7.9 cm, *P* < 0.001）。MG组的胸腺全切率较高（86.5% *vs* 72.7%, *P*=0.001），但两组患者的临床切除状态相似。

**2:** Masaoka分期为晚期时两组患者的相关资料 The specific analysis of late Masaoka stage patients between two groups

	MG (*n*=142)	non-MG (*n*=605)	*P*
Masaoka stage			0.128
Ⅲ	116 (81.7%)	458 (75.7%)	
Ⅳ	26(18.3%)	147 (24.3%)	
WHO classification			< 0.001
A	2 (1.4%)	14 (2.3%)	
AB	15 (10.6%)	38 (6.3%)	
B1	19 (13.4%)	29 (4.8%)	
B2	39 (27.5%)	71 (11.7%)	
B3	54 (38.0%)	154(25.5%)	
C	13 (9.2%)	273 (45.1%)	
Carcinoid	0 (0.0%)	26 (4.3%)	
Complete thymectomy	122 (86.5%)	434 (72.7%)	0.001
Tumor size	6.4	7.9	< 0.001
Resectability	104(73.2%)	400 (66.4%)	0.119
Recurrence	16(15.7%)	137 (31.7%)	0.001
注：本表得到版权所有者©2011-2016 Journal of Thoracic Disease复制许可。			

单因素分析提示MG、WHO分型、Masaoka分期、手术方式、化疗、放疗和临床切除结果是影响生存的因素（表 3）。然而在多因素分析中，*Cox*回归模型（表 4）的结果提示只有WHO分型、Masaoka分期、化疗和临床切除状态是独立的预后预测因子。

**3:** 总体患者的单因素分析 Univariate analyses in all patients

Factors	*P*
Gender (male *vs* female)	0.088
Age (> 50 yr *vs* < 50 yr)	0.289
MG (yes *vs* no)	0.038
Tumor size (> 5 cm *vs* < 5 cm)	0.459
WHO classification (A /AB or B1 or B2 or B3/C+NELL)	< 0.001
Masaoka's staging (Ⅰ/Ⅱ/Ⅲ/Ⅳ)	< 0.001
Approach of operation (thorascope *vs* open)	0.043
Thymectomy (incompletely *vs* completely)	0.041
Radiotherapy (no *vs* yes)	< 0.001
Chemotherapy (no *vs* yes)	< 0.001
Resectability (R0 *vs* R1+R2)	< 0.001
注：本表得到版权所有者©2011-2016 Journal of Thoracic Disease复制许可。	

**4:** 总体患者的多因素分析 Multivariate analyses in all patients

Factors	*P*	OR (95%CI)
MG (no *vs* yes)	0.967	1.016 (0.479, 2.157)
Gender (female *vs* male)	0.738	0.924 (0.580, 1.470)
WHO classification	0.012	
AB+B1+B2+B3 *vs* A	0.847	3335.39 (0, 2.521×10^38^)
C+NETT *vs* A	0.827	7582.361 (0, 5.732×10^38^)
Masaoka stage(Ⅳ/Ⅲ/Ⅱ/Ⅰ)	0.001	
Ⅱ *vs* Ⅰ	0.050	3.046 (1.002, 9.254)
Ⅲ *vs* Ⅰ	< 0.001	6.423 (2.489, 16.577)
Ⅳ *vs* Ⅰ	< 0.001	7.034 (2.416, 20.474)
Radiotherapy (yes *vs* no)	0.138	0.656 (0.376, 1.145)
Chemotherapy (yes *vs* no)	0.046	1.723 (1.009, 2.942)
Approach of operation (thorascope *vs* open)	0.655	1.401 (0.319, 6.147)
Tumor size (≥5 cm *vs* < 5 cm)	0.448	0.780 (0.411, 1.481)
Resectability (R0 *vs* R1+R2)	0.003	0.457 (0.273, 0.766)
Thymectomy (completely *vs* incompletely)	0.593	1.148 (0.692, 1.906)
NETT: Neuroendocrine tumors of the thymus. 注：本表得到版权所有者©2011-2016 Journal ofThoracic Disease复制许可。		

在Masaoka晚期的患者中，非MG组的术后复发率高于MG组（图 5，图 6）。

**5:**
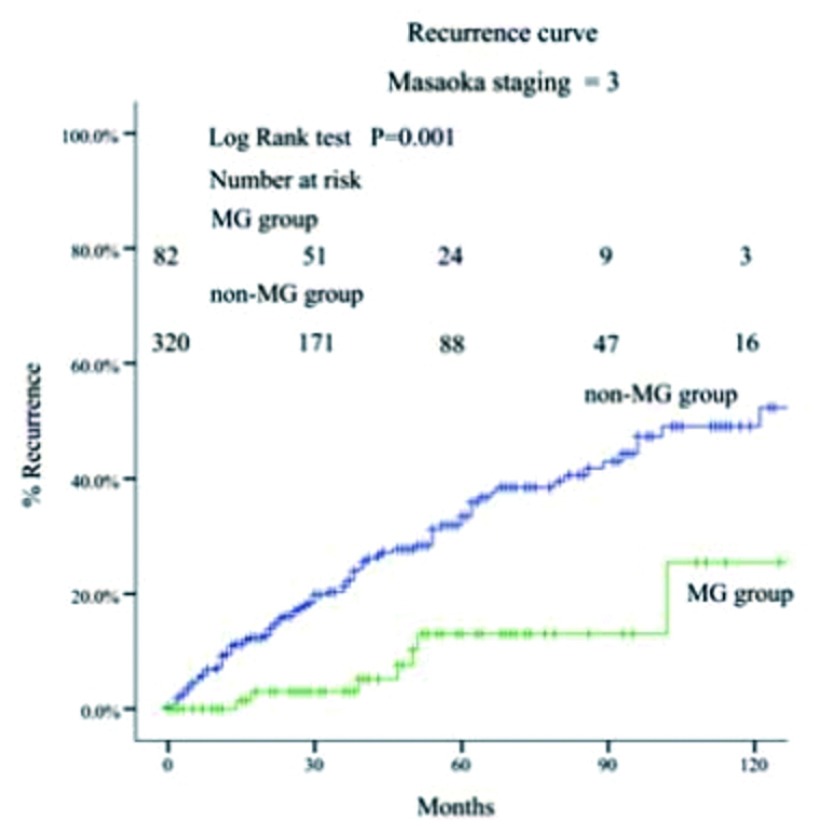
Masaoka晚期时两组患者的术后复发曲线 Comparison of tumor recurrence between myasthenia gravis (MG) group and non-MG group in advanced stage

**6:**
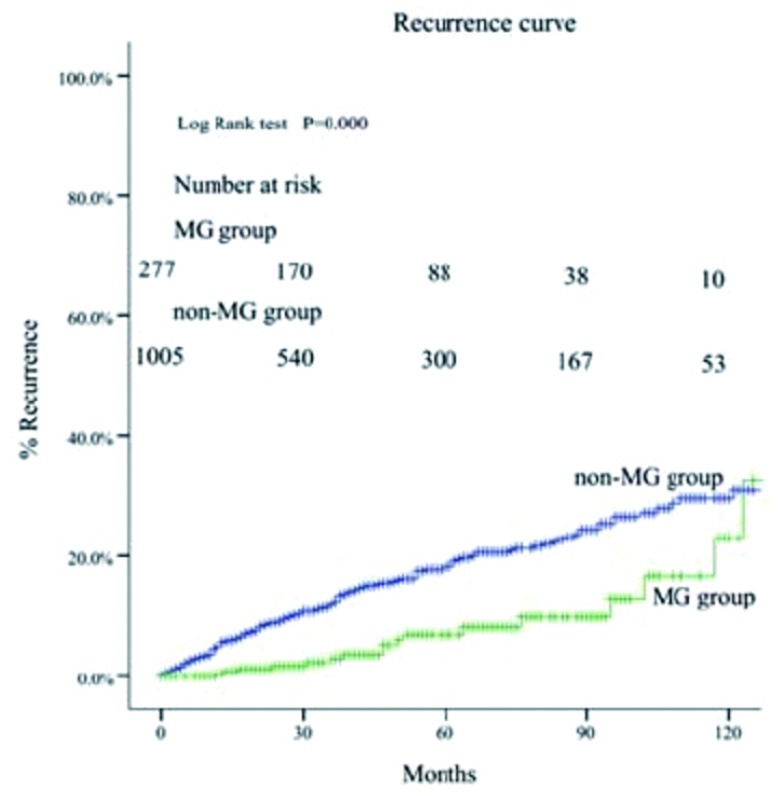
两组患者的术后复发曲线。 Comparison of tumor recurrence between myasthenia gravis (MG) group and non-MG group

## 3 讨论

当胸腺瘤合并重症肌无力时，与单纯胸腺瘤明显不同，不仅仅使围手术期的治疗复杂化，在某种程度上也改变了预后。关于胸腺瘤术后的预后，早期的研究结果与现今相比已是大相径庭^[2, 3, 5]^。最终本次多中心研究得到了明确的结论，重症肌无力对胸腺瘤患者的预后的确是有影响的。MG组患者的肿瘤分期相对较早，组织学上低度恶性（AB型和B型）的比例也较高，相比之下，单纯胸腺瘤组的患者被发现时多为进展期肿瘤从而导致病理分期显示出更差的趋势。MG的起病可能使得诊断及时并且能在第一时间发现肿瘤。作为多中心研究中独立的预后影响因子，总体的R0切除率在MG组中也明显高于单纯胸腺瘤组。上述的原因可能解释了为何重症肌无力的存在对胸腺瘤患者的长期预后带来了正面的影响。

一些文献^[6, 7]^报道了MG、肿瘤组织学特性和Masaoka分期之间的内在关系。Enrico Ruffini等提出了在肌无力患者中最常见两种类型，一种是Masaoka早期WHO A/A B型，另一种是Masaoka晚期WHO B型。他提到了MG对Masaoka分期和组织学分型的影响，在他的研究中提示只有Masaoka分期对总体生存率和无病生存率有预测性^[6]^。他的结论与我们本次多中心研究的结果相似，不过在ChART数据库的结果中，提示除了Masaoka分期之外，肿瘤组织学分型和临床切除状态也是预后的独立预测因子，并且MG似乎对这三个因素都带来了正面的影响。

重症肌无力对胸腺瘤患者预后的影响方式很奇特，虽然本次多因素分析的结果中MG并不是预后的独立预测因子，但是单因素研究的结果提示合并肌无力的患者其整体生存时间的确是延长的。进一步的研究发现MG对不同病理分期的患者产生的影响是不同的。理论上如果给予恰当的治疗与监护，重症肌无力通常并不致命，也不增加胸腺瘤手术的病死率，然而如果缺乏经验忽视MG的治疗，的确会增加围手术期肌无力危象或者呼吸相关并发症的发生。在本研究中，病理早期的患者中肌无力组的预后更差，可能就是因为术后肌无力相关并发症导致的死亡。但奇特的是，当胸腺瘤进入进展期后，肌无力组的生存优势变得尤其明显，这可能是其组织学分型的优势以及低复发率带来的益处。虽然肌无力增加了早期患者的死亡率，一定程度上抵消了它所带来的生存优势，但在单变量分析中还是在总体生存率上显示出其正面的影响。欣喜的是目前对肌无力的理解和治疗日趋成熟，进一步减少了肌无力相关并发症带来的死亡，并且对待合并肌无力的胸腺瘤时多学科合作也更加紧密，在将来的研究中这一类患者会显示出更良好的预后。

作为回顾性研究，ChART数据库中患者信息的缺失导致了大量病例被剔除，偏倚的发生也在所难免。另外，数据库中收集的患者跨度20年，在这段时间里，医疗技术的进展尤其是重症肌无力的治疗今非昔比，所以这些患者所接受的治疗基础并不一致，仍然需要前瞻性的研究来证实本研究的分析结果。

## References

[1] Maggi G, Casadio C, Cavallo A (1991). Thymoma: results of 241 operated cases. Ann Thorac Surg.

[2] Okumura M, Miyoshi S, Takeuchi Y (1999). Results of surgical treatment of thymomas with special reference to the involved organs. J Thorac Cardiovasc Surg.

[3] de Perrot M, Liu J, Bril V (2002). Prognostic significance of thymomas in patients with myasthenia grav is. Ann Thorac Surg.

[4] Maggi G, Casadio C, Cavallo A (1989). Thymectomy in myasthenia gravis. Results of 662 cases operated upon in 15 years. Eur J Cardiothorac Surg.

[5] Kondo K, Monden Y (2005). Thymoma and myasthenia gravis: a clinical study of 1, 089 patients from Japan. Ann Thorac Surg.

[6] Ruffini E, Filosso PL, Mossetti C (2011). Thymoma: inter-relationships among World Health Organization histolog y, Masaoka staging and myasthenia gravis and their independent prognostic significance: a single-centre experience. Eur J Cardiothorac Surg.

[7] Filosso PL, Venuta F, Oliaro A (2014). Thymoma and inter-relationships between clinical variables:amulticentre study in 537 patients. Eur J Cardiothorac Surg.

